# Raman Open Database: first interconnected Raman–X-ray diffraction open-access resource for material identification

**DOI:** 10.1107/S1600576719004229

**Published:** 2019-05-28

**Authors:** Yassine El Mendili, Antanas Vaitkus, Andrius Merkys, Saulius Gražulis, Daniel Chateigner, Fabrice Mathevet, Stéphanie Gascoin, Sebastien Petit, Jean-François Bardeau, Marco Zanatta, Maria Secchi, Gino Mariotto, Arun Kumar, Michele Cassetta, Luca Lutterotti, Evgeny Borovin, Beate Orberger, Patrick Simon, Bernard Hehlen, Monique Le Guen

**Affiliations:** a Normandie Université, CRISMAT-ENSICAEN, UMR6508 CNRS, Université de Caen Normandie, 6 Boulevard Maréchal Juin, 14050 Caen, France; b Vilnius University, Institute of Biotechnology, Saulėtekio av. 7, LT-10257 Vilnius, Lithuania; c Institut des Molécules et Matériaux du Mans, UMR6283 CNRS, Le Mans Université, Avenue Olivier Messiaen, 72085 Le Mans, France; d Department of Computer Science, University of Verona, Strada Le Grazie 15, 37134 Verona, Italy; e Department of Industrial Engineering, University of Trento, via Sommarive 9, 38123 Trento, Italy; f GEOPS-Paris Sud, Université Paris-Saclay, UMR8148 (CNRS-UPS), Bâtiment 504, 91405 Orsay, France; g CEMHTI, UPR CNRS 3079, Université d’Orléans, 1D Avenue de la Recherche Scientifique, 45071 Orléans Cedex 2, France; h Laboratoire Charles Coulomb, UMR5521 CNRS, Université Montpellier 2, Place Eugène Bataillon, 34095 Montpellier Cedex 5, France; i ERAMET, 1 Avenue Albert Einstein, 78190 Trappes, France

**Keywords:** Raman spectroscopy, open databases, combined Raman–X-ray diffraction, DDLm dictionary, CIF2

## Abstract

Detailed crystallographic information provided by X-ray diffraction (XRD) is complementary to molecular information provided by Raman spectroscopy. Accordingly, the combined use of these techniques allows the identification of an unknown compound without ambiguity. However, a full combination of Raman and XRD results requires an appropriate and reliable reference database with complete information. This is already available for XRD. The main objective of this paper is to introduce and describe the recently developed Raman Open Database (ROD, http://solsa.crystallography.net/rod). It comprises a collection of high-quality uncorrected Raman spectra. The novelty of this database is its interconnectedness with other open databases like the Crystallography Open Database (http://www.crystallography.net/cod and Theoretical Crystallography Open Database (http://www.crystallography.net/tcod/). The syntax adopted to format entries in the ROD is based on the worldwide recognized and used CIF format, which offers a simple way for data exchange, writing and description. ROD also uses JCAMP-DX files as an alternative format for submitted spectra. JCAMP-DX files are compatible to varying degrees with most commercial Raman software and can be read and edited using standard text editors.

## Abstract 2

The fully open character of the Raman Open Database makes this novel database freely and completely usable by anybody worldwide: academics, those working in industry and the general public.

## 1. Introduction   

Raman spectroscopy (RS) is a well known technique for the structural and compositional identification of materials in the solid, liquid or gas state. The analytical capabilities of Raman spectroscopy for both macro- and micrometric materials have made this technique the only appropriate one for many applications, *e.g.* where materials are very expensive or rare (Larkin, 2011 ▸). Recently, thanks to the development of surface enhanced Raman spectroscopy (SERS), RS has become an analytical tool with outstanding performance for biological and biomedical sciences (Mohs *et al.*, 2010 ▸; Nima *et al.*, 2014 ▸; Beermann *et al.*, 2009 ▸; Butler *et al.*, 2016 ▸). SERS offers an increase of several orders of magnitude in Raman intensity (up to 10^10^–10^14^), thus overcoming the traditional drawback of Raman scattering and allowing even single-molecule detection (Beermann *et al.*, 2009 ▸; Krug *et al.*, 1999 ▸; Rull *et al.*, 2017 ▸; Andreou *et al.*, 2015 ▸).

The increasing popularity of Raman spectroscopy stems from its easy sampling methods and its rapid response. Samples, in the form of powder, crystal, fiber, thin layer, gel, solution *etc.*, can be studied with a minimum of preparation. To make the technique more attractive, several attempts to develop miniaturized Raman systems for the analysis of surfaces have been made in recent years, and these paved the way, for example, to the exploration of the surface of Mars. Indeed, a Raman spectrometer was integrated as a part of the miniaturized system inside the ExoMars 2018 mission led by the European Space Agency and the Russian Roscosmos State Corporation (Rull *et al.*, 2017 ▸). The reason for using Raman spectroscopy in this project was to provide direct information on potential organic compounds that could be related to present or past signatures of life on Mars, as well as general mineralogical information for igneous, metamorphous and sedimentary processes, especially water-related geo-processes. To address these exobiological and geochemical issues, the project office selected a combination of different analytical methods, and RS is part of an analytical suite of four instruments (infrared spectroscopy, multispectral imager, organic molecule analyzer and Raman spectroscopy).

To maximize the information gathered from a particular material, RS and other analytical techniques, like IR and/or X-ray fluorescence (XRF) spectroscopies and X-ray diffraction (XRD), can be combined to complement each other. A combined system is being developed by the SOLSA project (http://www.solsa-mining.eu) led by a European Consortium within the EU-H2020 Raw Materials program. This project aims to build an expert system integrating for the first time sonic drilling and an automated on-line real-time mineralogical and chemical analysis with an off-line combined XRD–XRF–Raman analysis. An integrated and smart data architecture and software will convert the big data into actionable data (Duée *et al.*, 2017 ▸). The SOLSA expert system is designed for undestroyed drill cores and will be validated for nickel laterites. Such expert systems are in high demand from mining and metallurgical companies seeking to speed up exploration and mining and to define geo-metallurgical parameters for beneficiation. Nowadays, mineralogical analyses are only done by means of a single technique, such as hyperspectral imaging or XRF (Secchi *et al.*, 2018 ▸; Haest *et al.*, 2012 ▸; Roache *et al.*, 2011 ▸). The coupling of different analytical instruments is still a technological challenge in progress in our laboratories (Secchi *et al.*, 2018 ▸).

Raman spectroscopy and XRD are complementary analytical methods and are presented as a powerful tool to provide information related to chemical structures and functional groups of target species (Duée *et al.*, 2019 ▸; El Mendili *et al.*, 2012 ▸, 2016 ▸). Owing to their complementarity, each technique provides specific chemical information, and once coupled, comprehensive structural information at both long and short structural orders can be simultaneously collected without losing any important chemical information. In addition, RS overcomes the limitations of XRD, for example, when X-ray methods do not detect a signal (diluted species, weak scatters, unfavorable X-ray contrasts…). RS suffers also from some limitations, which are mainly those derived from the fluorescence emission induced in the samples under laser excitation. The complementarity of RS and XRD also lies in their different probed volumes. The former is usually restricted to micrometre-sized probes (particularly when optical microscopy is used), offers large phase detectability but lacks accurate quantification at the macroscale. By contrast, XRD with its relatively macroscopic probe better targets phase quantification but is far less sensitive to phases present in low amounts in the material volume.

Consequently, combined XRD–RS analyses together with Raman and XRD databases will offer an unprecedented capability to benefit from the complementary information from both techniques, allowing deeper understanding and description of materials. XRD–Raman analysis produces diffraction patterns and Raman spectra, which can be compared with open databases to give precise information about the crystal structure and the vibrational dynamics of the material examined. In the case of mixtures of compounds, the relative amounts of each compound can be quantified.

In this paper, the novel Raman Open Database (ROD; http://solsa.crystallography.net/rod) is introduced and described. It represents a collection of high-quality uncorrected Raman spectra and contains currently more than 1100 entries related to over 500 different phases. It is interrelated with other open databases like the Crystallography Open Database (COD; https://www.crystallography.net/cod). The fully open character of this novel database makes the ROD freely and completely usable by anybody worldwide, academics, those working in industry and the general public.

## 2. Results   

### 2.1. Current state of the ROD   

Currently, the database contains about 1100 entries in the standard CIF format with data related to over 500 different phases (more than a 1000 records in 10 months). Its development and success depend completely on future contributions of the community, by data upload or/and by giving help in improvements. Visit the ROD project web pages (http://solsa.crystallography.net/rod/) for more details.

The ROD web site has been designed similarly to that of the COD. The search tool and deposition subroutines have been adapted to Raman spectrum contents. A provisional version of a DDLm dictionary has been developed to describe the ontology of the Raman spectroscopy experiments (Spadaccini & Hall, 2012 ▸). The Advisory Board (AB) is under construction and examines applications from volunteers and experts of the field.

### 2.2. Content of the ROD database   

The ROD database is intended to handle experimental as well as theoretical Raman spectra. All types of materials (organic and inorganic materials, minerals, polymers, metals, ceramics, pigments, drugs, nuclear materials, hazardous materials…) will be collected to provide the largest possible set of single-phase spectra for the identification of materials. Experimental and simulated Raman data are accepted if they meet certain quality criteria and if enough experimental details are provided.

Simulated spectra are needed mainly for phases that cannot be produced with sufficient purity. However, since Raman spectrum calculations are still very time consuming, using first-principles approaches like density functional theory for instance, automated identification from structure files (like those accessible from the COD) is still not used routinely. Simulated spectra cannot provide exact information, in particular for phases including transition elements, and although these theoretical approaches are very useful, they cannot fully replace experimental data. Simulated data also provide good test cases of the models incorporated in the simulations when compared with experimental data. On the other hand, experimental data incorporate instrumental contributions, which can provide different Raman spectra of the same sample. This implies the collection of spectra with as many instrumental arrangements as possible for a given phase, in terms of incident wavelength, spectral resolution, optical detection, polarization state *etc*. In this respect, simulation also can provide fruitful complementarity. The ROD database can be used for phase identification provided these limitations are understood.

Supervised by the AB, the ROD provides access to only high-quality and reliable Raman data. Such data can come either from peer-reviewed publications or from personal data communications, which have been measured and analyzed by established Raman experts. Each entry in the ROD (associated with a unique ROD ID code) is associated with one .rod file, corresponding to one phase from one sample. For published data the entry then incorporates directly the publication data of the given phase/sample combinations. One publication can therefore give rise to several .rod files, and in each of these files the publication is carefully recorded. Furthermore, for personal communications, in each .rod file the measurement date, the authorship and the experimental conditions are recorded. It is then possible to track samples measured over time periods, under different instrumental configurations and resolutions, by different operators.

Such information is recorded through the on-line deposition process. Entry modifications by the initial depositor are allowed with comments, and are versioned automatically by the system.

All ROD data are openly available, either as individual .rod files from the search on-line form or as repositories. Data are released into the public domain.

### 2.3. The ROD Advisory Board   

The ROD is governed by the Advisory Board, which is a group of world-renowned Raman and programming specialists, the list of whom is visible on the ROD front page.

The AB members discuss and make all decisions regarding the scope, organization, quality criteria and development of the database, its scientific standards, maintenance *etc*. with the goal of creating a free open-access high-quality scientific database. A management mailing list is established for decisional and voting purposes.

This AB is not closed, and additional experts are welcome to apply, if they are willing to actively participate in aspects of evolution of the ROD, such as data upload, management, dictionary development and mirroring.

New AB members can be self-declared or invited by the AB. Typical acceptance requires a maximum of 15 days internal round discussion and vote. On the other hand, there is no need to be in the AB to upload data.

## 3. Discussion   

Open-access data repositories play an increasingly important role in the scientific community. The COD is one such resource, developed over the past 14 years, which has become the largest curated and validated open-access collection of small-molecule crystal structures (Gražulis *et al.*, 2012 ▸). Recently, a number of computer programs capable of automatically processing COD data sets have been developed, enabling various academic and industrial tasks (Gražulis *et al.*, 2015 ▸). In turn, this enabled the automated generation of structural formula descriptors and eased the establishment of cross-links between the COD and other open-access resources (Gražulis *et al.*, 2015 ▸). New strides have also been made in relating spectral data to their corresponding crystal structures.

The ROD is a very recent resource offered to the scientific community and is interrelated with the COD.

Several open-access libraries of experimental Raman spectra already exist. The most renowned and widely used is the RRUFF database (http://rruff.info/), dedicated to minerals, with more than 14 000 entries with high-resolution Raman spectra (Lafuente *et al.*, 2015 ▸).

Other smaller (in number of entries), freely accessible databases include the following:

(1) The Handbook of Raman Spectra for Geology (http://www.geologie-lyon.fr/Raman/) contains 73 mineral species.

(2) The Romanian Database of Raman Spectroscopy (http://rdrs.uaic.ro/) contains 72 individual mineral species (only carbonates and silicates).

(3) The Raman Spectroscopic Library of Natural and Synthetic Pigments (http://www.chem.ucl.ac.uk/resources/raman/index.html) contains Raman spectra of 64 common pigments.

(4) The Raman Spectra of Carbohydrates (http://www.models.life.ku.dk/~specarb/specarb.html) contains 26 Raman spectra of carbohydrates.

(5) The mineral Raman spectra of Parma University (http://www.fis.unipr.it/phevix/ramandb.php) has a few hundred entries.

(6) The Infrared & Raman Users Group (http://www.irug.org/search-spectral-database) contains 303 Raman spectra of different material classes.

(7) Calculated mineral Raman spectra are also available in the WURM project (http://www.wurm.info/) with 644 entries.

At first glance, it appears that all the previous databases unfortunately concern only a single range of materials. We also could not track (with a few exceptions) the original publications to which the available data sets belong. Furthermore, the instrumental characteristics are not described in sufficient detail (except for the Handbook of Raman Spectra for Geology). The combination of all these databases would be of great interest to complement the views offered by different Raman spectrometers, with diverse specifications, performance and measured ranges. For instance, laboratory-based instruments and portable and handheld instruments give different characteristics, and spectra obtained on one instrument cannot readily be compared with a library acquired on another.

Some poor-quality spectra without discernible vibration modes could also be found in some existing databases, which would prevent any corresponding phase identification in a compound. In addition, some Raman spectra appeared to be measured on multiphase samples. In some cases, ambiguities and contradictions can be present. For instance, spectra proposed for mackinawite, pyroxferroite and clinoferrosilite (RRUFF IDs R060388, R141080 and R141085, respectively) are similar, and could correspond equally well to the highly stable hematite (R060190), as proposed by several authors (El Mendili *et al.*, 2015 ▸; Hanesch, 2009 ▸). But more importantly, none of the previous databases allows the extraction of the whole data set for separate analysis, nor are they internally developed for interoperability. Since the ROD is developed on the same model as the COD, this is directly possible. In the ROD, the strategy is to perform Raman measurements and collect published Raman data from single phases, with a variety of Raman instruments (microscope or fiber, portable…), including different wavelengths and resolutions. Spectral quality validation is carried out one by one on personal communications, with verification of vibration mode assignments, and where possible it is ensured that the associated materials and publications are traceable. All the spectra will be analyzed and validated by world-renowned Raman specialists belonging to the AB.

In summary the ROD has been developed and is under continuous development with high-quality Raman spectra, interconnected to structural data from the COD. The CIF2 and the JCAMP-DX formats have been adopted for their suitability of storing scientific data with appropriate versioning and interconnectivity. This curated set of Raman spectra is freely available to all individuals, institutions and companies, and will collect and accept all equal-quality spectra from other sources. The actual ROD contains both experimental and theoretical spectra. Finally, the ROD is not restricted to crystalline phases and contains all classes of materials (minerals and pigments, glasses, organic and inorganic materials, polymers, metals, ceramics, pigments, drugs, nuclear materials, hazardous materials…).

## 4. Methodology   

### 4.1. The CIF format   

All published and personal communication (reference) spectral data are stored in the ROD using CIF as the homogeneous data carrier format, as already used in the COD for structural data. CIFs are written according to the CIF 2.0 file specifications (Bernstein *et al.*, 2016 ▸) and given the extension .rod. This human-readable text-file format allows the definition of all experimental and data set components in a simple way, with unambiguous descriptions. This involved the development of a Raman-spectroscopy-oriented DDLm dictionary, *i.e.* the Raman dictionary. Each experimental (from the excitation source to the detecting system, via all optical paths) and measurement component resulting in the corresponding sample characteristics is defined in the Raman dictionary. This latter development, part of the SOLSA project, allows interoperability between elemental and short- and long-range ordering characteristics of the phases, as probed by XRF, Raman–IR and XRD probing techniques, respectively, in a uniform computer-readable way. The latest version of the Raman dictionary is available online (http://solsa.crystallography.net/rod/cif/dictionaries/cif_raman.dic).

After a .rod file is constructed, it is submitted to the database site, which checks for syntax. When a syntax check is successful, an initial ROD ID is assigned to the first structure. All data are split into separate CIFs, one spectrum per CIF, and numbered sequentially.

When all the checks are passed, each resulting ROD CIF is recorded in the ROD MySQL database, and the file immediately becomes available on the web.

### 4.2. ROD file identification   

Each structure recorded in the ROD receives a unique seven-digit number, called a ROD ID. A ROD ID identifies Raman spectra measured on a single phase at a given time. As a rule, the ROD accepts duplicate structures. For instance, if two spectra of the same phase are published in two different peer-reviewed papers, both can be deposited to the ROD and receive different ROD IDs. Indeed, all separate information from the same phase is very valuable for cross comparisons. For all personal communications to the ROD (spectra that are not published and are not being planned to be published), since Raman spectra strongly vary with instrumental conditions, and eventually under the same experimental conditions might vary over time (because of aging and uncontrolled parameters), all newly added .rod files require the measurement date. This is the purpose of the data item _raman_measurement.datetime_initiated, which stores the date/time when the experiment was started. It could be used as one of the criteria to differentiate between similar measurements and even potentially avoid duplicates.

### 4.3. Web design and server infrastructure   

**Figure 1:**
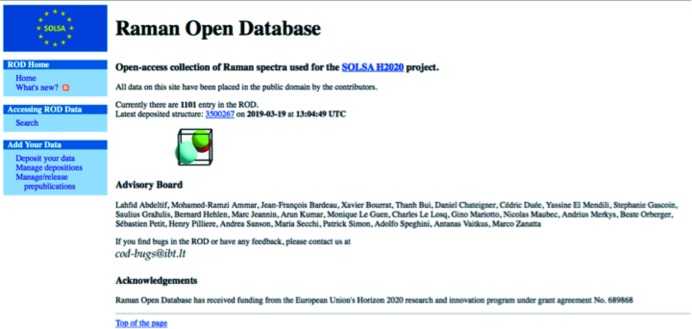
Snapshot of the ROD home page (3 July 2018).

A web interface was created for searching the information contained in the ROD files. We use an Apache web server running on a Linux platform. The server is regularly maintained and upgraded to ensure performance and availability. In the future, mirrors are foreseen to ensure data availability over time, alongside the COD actual sites (Fig. 1 ▸). There is no restriction to data access, which can be reused in any further software and databases. All data are placed in the public domain.

**Figure 2:**
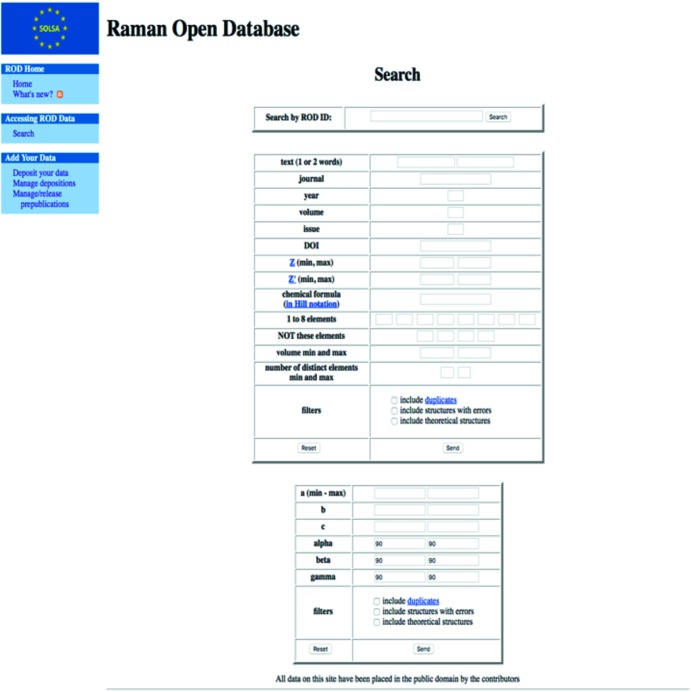
Snapshot of the ROD search page.

The site provides general information about the ROD, the Raman dictionary, and references to the CIF file structure, syntax and core dictionary. Moreover it offers the possibility of searching files (Fig. 2 ▸) according to ROD ID, phase name and/or the elements it contains, unit-cell characteristics (cell parameters and unit-cell volume ranges), chemical formula, or bibliographic details. Several other filters are available and will be developed.

### 4.4. Composition of ROD files   

As for all CIF-related databases, all definitions from the core CIF dictionary remain available and can be included in the .rod files and are accordingly interpreted. The composition of a ROD file can be divided into nine, not all mandatory, categories.

**Bibliography**

This category represents the bibliographic references of the main source, with for example author names, publication or book title, journal name, page/article numbers, volume, year, and DOI.

**Sample details**

Records information on the phase under study and the origin of the analyzed sample:

(*a*) Composition and chemical formula of the phase, its cell parameters and space group, and, if possible, any further useful details (*e.g.* mineral name, color).

(*b*) Sample origin (*e.g.* the place of discovery for minerals or the actual source of a natural product, mine).

**Raman determination**

Indicates if the recorded data come from measurement or from simulations (from first-principles calculations for instance).

**Raman theoretical spectrum**

If the Raman spectrum comes from simulations, the used items record the details of the Raman spectrum predictions together with the analysis methods used to predict the active Raman bands.

**Complementary observation**

Details the methods used to approve the collected Raman spectrum data. For instance, X-ray diffraction, energy dispersive X-ray spectroscopy, X-ray fluorescence, ion mass spectrometry, infrared spectroscopy, near-infrared spectroscopy, Mössbauer spectroscopy, chemical analysis….

**Instrument characterization**

*Instrument details*

This category specifies the Raman measurement device information. The following information is used to characterize the Raman equipment:

(*a*) Physical location of the device at the time of measurement.

(*b*) Name of the company that constructed the spectrometer.

(*c*) Model of the measuring instrument.

(*d*) Type of optics used to focus the primary laser beam onto the sample and/or to collect the scattered radiation.

(*e*) Types of used microscope (microRaman configuration).

(*f*) Magnification of the microscope objective.

(*g*) Numerical aperture of the microscope.

(*h*) Type of exciting laser used.

(*i*) Exciting wavelength used for the measurement (in nm).

(*j*) Monochromator configuration.

(*k*) Measurement device resolution (in cm^−1^).

(*l*) Beam power on the sample (in mW).

(*m*) Polarization.

When using polarized measurements, it is important to keep track of the polarization conditions so that the results can be consistently interpreted. To describe the propagation and polarization directions, we adopt the commonly used Porto notation (Damen *et al.*, 1966 ▸). This notation expresses the orientation of the crystal frame with respect to the polarization directions of the laser for both exciting and analyzed beams. The notation of Porto, for Raman scattering processes, consists of four letters: A(BC)D:

A = incident light propagation direction, wavevector **k**_i_.

B = incident light polarization direction **E**_i_.

C = scattered light polarization direction **E**_s_.

D = scattered light propagation direction, wavevector **k**_s_.

**Figure 3:**
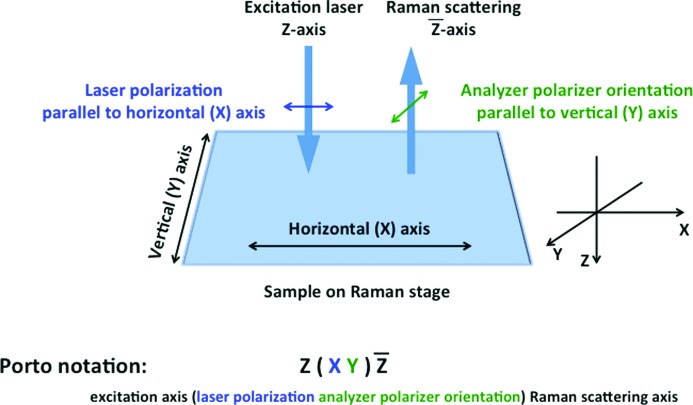
Orientation of Raman microscope polarization axes at the sample and the corresponding Porto notation for non-oriented samples.

For instance (Fig. 3 ▸), Y(XZ)X means that the incident light propagates along the *Y* axis of the spectrometer reference frame, while the scattered light is detected along *X*, with the incident light polarized along *X* while the scattered light is analyzed along *Z*.

(*n*) Spot size on the sample (in µm).

(*o*) Diffraction grating (lines per mm).

*Instrument calibration*

Details of the reference sample and procedures used for the calibration of the measuring device.

**COD database entry**

Reference code relating to the COD entry to which the actual ROD phase belongs (see Section 4.6 for an example). Since the ROD data are cross-linked to structural data of the COD, some of the phase parameters (*e.g.* unit cell, space group) are automatically harvested from the COD to the .rod file and do not need to be entered by the user.

**Experimental details**

This category specifies the overall spectral measurement information.

*Experimental environment*

(*a*) Description of the sample environment.

(*b*) Temperature at which the sample was measured (unit K).

(*c*) Pressure at which the sample was measured (unit kPa).

*Analysis details*

(*a*) Description of the background subtraction that has been applied.

(*b*) Description of the baseline correction that has been applied.

(*c*) Total time spent for the acquisition of the Raman spectrum (unit s).

(*d*) Date and time (timestamp) recorded at the start of the measurement.

*Experimental data*

This is the Raman spectrum data set, as a two-column format, for the raw (uncorrected by any means) intensity [in arbitrary units (a.u.)] and the wavenumber (in cm^−1^).

**Template for .rod file construction**

Preparing .rod files can be tedious for beginners, and time consuming. In order to ease the .rod construction process, we have developed a web form (http://www.crystallography.net/cod/cif-template) to generate the ROD files automatically. The web form is created automatically from the current Raman CIF dictionary and thus contains entry fields for all data items that are available for a valid ROD file. Code snippets in JavaScript are used to check the most important constraints on the input values (such as the presence of at least one value in a data table, which is a syntactic requirement for a ROD CIF). Large data tables (most importantly, spectral data) can be pasted into the input text field directly from the output provided by any spectrometer. From the user-provided input values a syntactically correct and valid ROD file is generated, which can then be directly uploaded to the ROD deposition system.

**Minimum requirements**

The ROD Advisory Board compiled a list of criteria that are checked for in the input .rod files before inclusion into the ROD database. The following items are considered as the minimum information required before a .rod file can be accepted:

(i) High-resolution spectra.

(ii) Raman shifts and intensities.

(iii) Authorship and other bibliographic data. Authorship (personal communication) or complete bibliographic data (published paper).

(iv) Incident wavelength.

(v) Sample state (solid, liquid *etc*.).

(vi) Chemical formula.

(vii) Polarization.

(viii) Sample description and provenance (strongly recommended); if this field is left blank, the user means ‘unknown’.

(ix) Laser power.

(x) Spectrometer model.

(xi) Objective type.

(xii) Measurement resolution (should be present), or any other parameters helping its calculation.

(xiii) Experiment duration (should be present). This is the total time spent on one spectrum.

(xiv) Link to the COD. The best (and simplest) case is when the same sample is measured both with Raman and XRD. In this case, the user should link or upload the actual phase(s) from the COD. Otherwise, if no XRD is available the simplest choice is to select a ‘best candidate’ from the COD. This has to be considered as a suggestion and clearly identified. The third case is, of course, no best candidate and ‘unknown’. The methods of linking ROD entries to COD entries are described below.

The following apply additionally to personal communications:

(xv) Sample description and provenance.

(xvi) Experiment timestamps.

The ROD data quality criteria are given at http://wiki.crystallography.net/rod/data_quality_criteria/. These minimum requirements will be updated according to discussions and decisions of the members of the ROD Advisory Board.

### 4.5. ROD deposition procedure   

On the ROD front page a ‘Deposit your data’ menu is available. Anybody can upload data provided the above-mentioned minimum requirements are fulfilled by the files to be uploaded. Here is a short tutorial on how to use it:

First create your user account:

(1) Click the ‘Deposit your data’ link on the left side of the page.

(2) Click the ‘Sign up’ link.

(3) Follow the instructions on the page and fill in your registration data.

Then deposit your data:

(1) Click on ‘Deposit your data’.

(2) Choose the deposition type.

(*a*) Choose ‘Already published data’ if the data were published in a journal.

(*b*) Choose ‘Personal communication to ROD’ if your data were not published anywhere.

(3) Type in your data which will be used to create your username.

(4) Type in your login data and click ‘Begin deposition’.

(5) Click the ‘Browse…’ button and select the .rod file or a zip archive of .rod files that you would like to upload. JCAMP-DX is also accepted.

(6) Press the ‘Validate’ button. If there are any problems with your .rod file(s) you will be prompted to correct the errors. If you are unsure how to correct them, please e-mail us the error message and we will do our best to explain how to correct it.

(7) If your file is successfully validated you will be taken to a new window where you can finish your deposition. You should click the ‘Deposit all valid files to the ROD’ button or ‘Deposit to the ROD’ button for each individual file.

Syntactic errors, if any, must be corrected manually.

### 4.6. Relating ROD data to other online resources   

Raman spectroscopy data are useful on their own, but they become even more valuable when combined with additional facts about the measured material. The rise of the internet paired with modern advances in computing has made this task immeasurably easier by providing access to the vast sea of human knowledge in the form of online resources. However, it has also brought on the challenge of establishing relationships between those resources. This issue is addressed in the ROD by providing a set of CIF data items capable of describing links between different data resources in a way that is easily understood both by humans and by computers.

In order to establish a link between an entry in the ROD and an external resource, appropriate CIF data values must be placed in the ROD file. The minimal set of information required to describe such a relationship consists of the name of the resource and the unique identifier of the related entry belonging to that resource. The provision of additional information such as the explicit URI of the related entry and the description of the relationship, though not mandatory, is strongly encouraged. An example of a CIF data loop follows, describing a relationship between the ROD entry storing this loop and the COD entry 7228110. The description states that the entries are related on the grounds of the RS and XRD experimental data being acquired by measuring the same sample:

loop_

rod_related_entry.id

rod_related_entry.database

rod_related_entry.code

rod_related_entry.relation

1 COD 7228110 same_sample

The described method allows one to establish any number of relationships from the ROD to external resources as well as between the ROD entries themselves. A more detailed description of the data items and additional usage examples can be found in the CIF_ROD dictionary.

### 4.7. Further developments   

**Figure 4:**
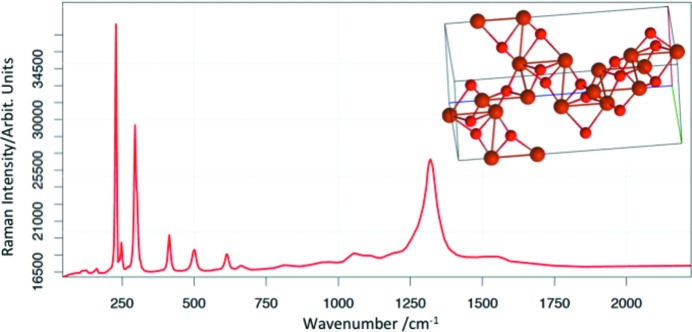
Raman spectrum and related crystal structure of hematite. ROD ID 1000001, COD ID 1546383.

The ROD is under continuous evolution, both in terms of quantity and quality of entries, and to ease implementation of new spectra and programming. One of the latest advances (Fig. 4 ▸) is the implementation of the phase structure from the COD on the experimental spectrum. This COD to ROD interoperability will be pushed further in the future.

## 5. Summary   

The Raman Open Database has been developed and is in continuous evolution with high-quality Raman spectra, interconnected to structural data from the Crystallography Open Database. The CIF2 format has been adopted for its suitability of storing scientific data with appropriate versioning and interconnectivity. This curated set of Raman spectra is freely available to all individuals, institutions and companies, and will collect and accept all equal-quality spectra from other sources. It is not restricted to crystalline phases, though at this stage it is skewed towards them.
